# Exploring the views and experiences of people recovering from a stroke about a new text message intervention to promote physical activity after rehabilitation—Keeping Active with Texting After Stroke: A qualitative study

**DOI:** 10.1111/hex.13809

**Published:** 2023-07-06

**Authors:** Albert Farre, Jacqui H. Morris, Linda Irvine, Stephan U. Dombrowski, Jenna P. Breckenridge, Gozde Ozakinci, Thérèse Lebedis, Claire Jones

**Affiliations:** ^1^ School of Health Sciences University of Dundee Dundee UK; ^2^ Faculty of Kinesiology University of New Brunswick Fredericton New Brunswick Canada; ^3^ Division of Psychology, Faculty of Natural Sciences University of Stirling Stirling UK; ^4^ NHS Grampian, Woodend Hospital Aberdeen UK; ^5^ Health Informatics Centre School of Medicine University of Dundee Dundee UK

**Keywords:** physical activity, qualitative research, stroke rehabilitation, text messaging

## Abstract

**Background:**

Participating in exercise following a stroke is essential for recovery. When community‐based rehabilitation services end, some people struggle to remain active. We codesigned Keeping Active with Texting After Stroke (KATS), a text message intervention to support home‐based, self‐directed plans to continue exercising. KATS delivers a series of automated text messages over a 12‐week period from the point of discharge from National Health Service‐funded therapy. The aim of this study was to explore the views and experiences of the first cohort of participants to complete the KATS intervention about the meaning, engagement, workability and worth of the intervention.

**Methods:**

We undertook a qualitative study, theoretically informed by Normalisation Process Theory. We conducted semi‐structured telephone interviews with people with stroke from two Health Boards in Scotland. Data collection took place over two phases, with each participant being interviewed twice: first, halfway through intervention delivery (Week 6) and then again at the end of the intervention (Week 12). All interviews were audio‐recorded, transcribed and analysed thematically.

**Results:**

A total of 24 interviews were conducted with 12 participants. Our findings were organised around four overarching analytical themes: (1) making sense of KATS: timing and complementarity in the rehabilitation journey; (2) engaging with KATS: connection and identification with others; (3) making KATS work: flexibility and tailorable guidance; (4) appraising the worth of KATS: encouragement and friendliness. Participants differentiated KATS from current rehabilitation practice, finding it relevant, fitting and worthwhile. Variations were reported in engagement with behaviour change techniques, but participants were able to tailor KATS use, making it work for them in different ways.

**Conclusions:**

Perceived benefits went beyond promoting physical activity, including feeling supported and connected. Future research will test the effectiveness of KATS in promoting physical activity and explore any associations with relevant social and emotional secondary outcomes.

**Patient or Public Contribution:**

A research funding proposal was developed in collaboration with five people with stroke and three spouses. After securing funding, six people with stroke were invited to join the project's Collaborative Working Group, alongside health professionals and stroke rehabilitation experts, to codevelop the intervention and support the feasibility study.

## 1 INTRODUCTION

In the United Kingdom and worldwide, stroke is among the top leading causes of death and disability combined, with the number of people living with stroke globally having almost doubled over the last 30 years.^1^ Participating in exercise and physical activity following stroke contributes to substantial health benefits. The direct physical benefits of exercise can support recovery through the improvement of walking ability, balance and fitness.^2^ In addition to the physical benefits of exercise, regular physical activity can also improve health‐related quality of life, reduce poststroke fatigue, enhance social participation and help to restore independence.^3^

However, evidence suggests that physical activity levels after stroke are low and further decline over time,^4^ with stroke survivors often experiencing physical deconditioning and leading sedentary lifestyles.^3^ This may be due to a wide range of factors both directly and indirectly related to stroke (e.g., prestroke physical inactivity and sedentary lifestyles, direct neurological effects of stroke which can reduce the muscle mass available for activation, presence of comorbid conditions) resulting in few people with stroke meeting recommended levels of physical activity. Therefore, finding effective ways to support people to become and remain active after stroke is critical.

Many people with stroke in the United Kingdom receive physiotherapy and occupational therapy at home following discharge from intensive in‐patient‐based rehabilitation.^5^ When this community rehabilitation ends, some people feel there is a gap in support provided and still struggle to remain active.^6^ The structured exercise programmes, guided by therapists, must be replaced by self‐directed plans to continue exercising and increase physical activity,^7^ which can be challenging for many.^8^, ^9^

Text message‐based interventions have the potential to support and improve home‐based, self‐directed plans to continue exercising when community rehabilitation ends, or when community rehabilitation services are not available (e.g., in countries or communities where access to basic rehabilitation services may be lacking).

Research to date has shown promising effects on increasing physical activity in general populations,^10^ however, interventions for people with stroke have yet to be fully tested. Pilot studies have reported the potential use of text message interventions for people with stroke, but their use has been limited and for diverse purposes: The STROKEWALK study delivered instructional text messages to promote regular walking and functional leg exercises over 3 months^11^; the iVERVE intervention used text messages as part of a self‐management programme to support goal attainment for recovery after stroke and in secondary prevention after stroke^12^; and a text message reminder based intervention, which was part of a family‐centred intervention, sought to support participation in daily activities.^13^, ^14^ These studies demonstrated that people with stroke can use text messaging as an intervention, although none specifically focused on using behaviour change strategies to support continuity with rehabilitation whilst facilitating the transition to active living after rehabilitation.

To provide continuity beyond formal rehabilitation and to help people with stroke to be physically active at the end of rehabilitation, we codesigned a novel text message intervention, the ‘Keeping Active with Texting After Stroke’ (KATS) intervention. The intervention and its development is described in detail elsewhere,^15^ but briefly, we used a multistage iterative process to codevelop a theoretically informed text message intervention in collaboration with people with stroke, health professionals and experts in the field of stroke rehabilitation who were invited to join the study's Collaborative Working Group.^16^ Key contributions from people with stroke and the Collaborative Working Group throughout the codevelopment process included: assessment of mobile phone use following stroke; identification of current needs and gaps; design of intervention goals; design of message contents and message delivery patterns; acceptability assessment; revision and refinement of messages. The intervention was designed to dovetail with community rehabilitation services after a stroke, to provide support and continuity at a time when many people with a stroke feel vulnerable. It was intended to enhance motivation, combat feelings of abandonment postrehabilitation and support the uptake and maintenance of physical activity and recovery‐specific exercises. The text messages were designed to provide support for goal setting, planning and self‐monitoring of physical/recovery activities and exercises. The intervention was theoretically informed by the Health Action Process Approach^17^ and used a range of established behaviour change techniques^18^ to increase motivation and provide support for people with stroke to be physically active.

An ongoing feasibility study was designed to test and refine the KATS intervention ready for evaluation in a future randomised controlled trial. We undertook a qualitative study to explore the views and experiences of the first cohort of participants to complete the KATS intervention as part of their participation in the intervention's feasibility study. This qualitative study was informed by Normalisation Process Theory (NPT)^19^—a sociological theory which explains the processes involved in implementing and/or making a new intervention work in practice to allow for the intervention to become ‘normalised’ or embedded in individuals/groups everyday practices. Four core constructs describe generative mechanisms that facilitate normalisation: coherence (work to make sense of an intervention), cognitive participation (work to engage with an intervention), collective action (work to enable an intervention to happen) and reflexive monitoring (work to appraise an intervention).

The aim of this study was to explore the views and experiences of the first cohort of people with stroke to complete the KATS intervention about the meaning, engagement, workability and worth of the intervention.

## 2 METHODS

We undertook a qualitative study, theoretically informed by NPT, using semi‐structured telephone interviews with people with stroke from two Health Boards in Scotland. Ethical approval was granted by the North of Scotland Research Ethics Service (21/NS/0028).

The KATS intervention^15^ comprised 95 text messages delivered to participants over a period of 12 weeks. Participants received at least one message every day. The first week was used to foster interest and engagement. Messages then followed a sequence to address and illustrate the process of behaviour change to increase physical activity. Messages used conversational, informal language to encourage engagement. Participants were advised that, while they were welcome to respond to any of the text messages, the KATS messaging system did not allow for the research team to reply to any of their responses. Some messages included pseudonymised quotes and examples from people with stroke who had participated in the intervention development process, and from participants in our previous studies.^20^, ^21^ These messages modelled behaviours and provided encouragement. Some messages were personalised to include participants' names. Text messages were delivered by an automated computer system which was programmed to send the messages to participants' mobile phones in a predetermined sequence. The software tool for delivery was developed by the Health Informatics Centre at the University of Dundee (C. J.). Participants were provided with a calendar (to facilitate recording of daily activities and reflection on progress) and a handbook (to reinforce key components of the intervention, and to provide information and signposting to online resources offering exercises for people who have had a stroke). At the end of the 12‐week intervention participants received a £20 gift voucher.

Data collection took place over two phases between July and November 2021, using semi‐structured telephone interviews, with each participant being interviewed twice: First, at 6 weeks postrecruitment (halfway through intervention delivery) and again at the end of the KATS intervention at Week 12. This was to enable the exploration of experiences of the intervention ‘in use’ alongside perceptions at the end of the intervention cycle. The choice of conducting telephone interviews, rather than using virtual platforms, was chosen to minimise the potential impact of digital literacy, or stroke‐related problems which can affect the ability to use digital technologies, as barriers to participation. Interviews were conducted by a female nonclinical university‐based researcher (L. I.) with extensive experience in intervention studies, including intervention development and feasibility testing studies using participatory and qualitative methods.

All participants from the first cohort to complete the KATS intervention as part of their participation in the intervention's feasibility study were invited and took part in this qualitative study. Recruitment for the feasibility study was undertaken in collaboration with staff from stroke rehabilitation services in two Health Boards in Scotland, who identified patients receiving rehabilitation and invited them to take part in the study. Interested patients signed an expression of interest form, which gave the research team permission to contact them when community rehabilitation was nearing completion. The research team did not have any prior relationship with the study participants. Potential participants were given general information about the research team and detailed information about the study and their potential participation at the point of being contacted by the research team. Informed consent was obtained using audio‐recorded telephone conversations, as face‐to‐face contact was not permitted due to COVID‐19 pandemic restrictions. A copy of the consent form signed and dated by the researcher was sent to the participant by email or post. Suitable times were arranged with all participants to collect data on participant characteristics before the start of the intervention. Participants were characterised by age, sex, time since stroke, whether they lived alone or not, sociodemographic category^22^ (Scottish Index of Multiple Deprivation [SIMD]) and Modified Rankin Scale^23^ to provide an assessment of disability/dependence. Times for telephone interviews were agreed at 6 and 12 weeks after the start of the intervention. All telephone interviews were audio‐recorded and transcribed. Interview topic guides were informed by NPT^19^ in combination with topics/prompts suggested by the larger feasibility study's Collaborative Working Group.^16^

Interview data were managed using NVivo software and analysed using thematic analysis^24^ theoretically informed by NPT.^19^ Data were initially open coded by the researcher who conducted the interviews (L. I.). To address any potential researcher bias or assumptions that might have impacted the analysis, the initial coding was critically reviewed by two other researchers (A. F. and J. M.). An agreed descriptive coding framework was developed, which was then revised and refined by the research team as coding proceeded and new data were collected and added to the data set. Descriptively coded data were then mapped against NPT constructs to inform the development of analytical themes (A. F.) which were critically reviewed (L. I. and J. M.) and further discussed and refined in group data analysis sessions until the findings were established. These sessions included regular data analysis meetings between the three researchers primarily undertaking data analysis tasks (L. I., A. F. and J. M.) as well as feedback sessions with the wider research team and the study's Collaborative Working Group, both of which included people with stroke.

## 3 RESULTS

A total of 24 interviews were conducted with 12 participants (Table 1). Each participant took part in two interviews, the first one halfway through intervention delivery (Week 6) and then again at the end of the KATS intervention (Week 12). Nine participants were male and three were female. Their ages ranged from 31 to 74 years (median 61 years). Three participants lived alone. There was representation across all sociodemographic categories of the SIMD.^22^ The time since stroke ranged from 5 to 184 weeks (median: 57 weeks). Participants self‐assessed their degree of disability/dependence using the Modified Rankin Scale,^23^ with scores ranging between 1 and 4 (maximum 5) and the majority of participants scoring 3 on the scale.

**Table 1:** Summary of participants' sociodemographic characteristics.

Characteristics	*N* = 12
	*n*
Sex	
Male	9
Female	3
Age	
<60 years	5
60–65 years	2
>65 years	5
Living arrangements	
Lives with partner/family	9
Lives alone	3
Scottish Index of Multiple Deprivation	
1–2 (Most disadvantaged)	4
3–4	6
5 (Least disadvantaged)	2
Time since stroke	
<1 year	5
1–2 years	5
>2 years	2
Modified Rankin Score (self‐assessed)	
1 (No significant disability)	2
2	1
3	8
4	1
5 (Severe disability)	0

Below we describe our main findings, organised around four overarching analytical themes, which were informed by the four core constructs of NPT (Table 2).

**Table 2:** Structure and organisation of themes.

Main descriptive themes	NPT constructs	Overarching analytical themes
Timing of intervention in the rehabilitation journey.	Coherence	*Making sense of KATS: timing and complementarity in the rehabilitation journey*.
Complementarity of KATS with existing guidance.		
Perceived connection addresses sense of isolation.	Cognitive participation	*Engaging with KATS: connection and identification with others*.
Identifying with quotes from others in a similar situation.		
Allowing flexibility to engage at different levels.	Collective action	*Making KATS work: flexibility and tailorable guidance*.
Offering guidance that can be adapted to a range of activities.		
Messages perceived as helpful and encouraging.	Reflexive monitoring	*Appraising the worth of KATS: encouragement and friendliness*.
Language and content perceived as accessible and friendly.		

Abbreviations: KATS, Keeping Active with Texting After Stroke; NPT, Normalisation Process Theory.

### 3.1 Making sense of KATS: Timing and complementarity in the rehabilitation journey

Participants perceived the KATS intervention as both relevant and fitting based on their rehabilitation journeys.

The perceived relevance of the KATS intervention was particularly driven by the timing of the intervention, which followed on from the point of discharge from National Health Service (NHS) funded therapy, a difficult time in the rehabilitation journey that some associated with a feeling of abandonment:

When [rehab centre] was finishing you have the slight feeling of being abandoned, you know. I know that wasn't the intention at all, but having the KATS study following on from that helped to make me feel I was still being kept in the loop and considered. So, I think the timing was probably pretty good, actually. (GW1007, male, 70 years—Week 12 interview)

I think it just follows on nicely because it started almost immediately when the physio stopped coming to the house. I don't think I would have seen the point in getting messages from you lads when the physios were still coming here. (TW1003, male, 69 years—Week 6 interview)

Some participants noted that an earlier starting point for the intervention might have suited them better:

I would say that the study would have been more useful if I had done it early—maybe as soon as I got out of hospital… Yes, I would say at the beginning of the supported discharge, so, I think this alongside supported discharge. (GW1001, male, 31 years—Week 6 interview)

The perceived fit of the KATS intervention was particularly driven by the consistency and complementarity of the intervention with the recommendations they had received from their therapists at the point of discharge from rehabilitation. In this context, KATS was seen as an element of continuity from services and additional support to enact recommendations and meet the expectations of rehabilitation:

[the proposed activities] were very much along a similar sort of pattern. (TW1004, male, 61 years—Week 6 interview)

[with the information and content of your messages] You're also crediting the girls [NHS physiotherapists] who came every day for those 6 weeks [of NHS funded therapy] because they were saying the things that you're saying in these texts, they were saying those things to me. There is no point in doing 6 weeks of these girls [NHS physiotherapists] coming and then just ignoring what they said. What you were saying in the texts was exactly what they were saying. (GW1008, male, 67 years—Week 6 interview)

### 3.2 Engaging with KATS: Connection and identification with others

The relevance and fit of the KATS intervention were further emphasised by the impact of COVID‐19 pandemic restrictions, which brought about an increased sense of isolation among participants:

You see, your messages—I know it sounds crazy, but your messages are like, when I'm alone and sitting on my own, it's giving me purpose and a reason to try, if you know what I mean. I could sit here and watch TV and do nothing. (TW1010, male, 57 years—Week 6 interview)

I was a bit stir crazy just sitting in and what have you, but the messages would help me to get going and what have you. (…) When I look back now, and knew that your texts were coming through, yes, it was like somebody was here, no knocking on my door, no face‐to‐face, but I always knew somebody would be there and I'm not the only one in the world with the problems. (GW1002, female, 60 years—Week 12 interview).

In this context, it became apparent from some participants' experiences that the nature and content of the KATS messages mattered as much to them as receiving text messages did:

Well, that's how I feel, that there's somebody out there that cares, you know—I know it's text messages, but it's like when your text message comes through, I'm like, somebody cares, somebody is thinking of me… So, yes, the messages spur you on and give you challenges. (GW1002, female, 60 years—Week 6 interview)

Sometimes it was just the actual message that was helpful, because if you're not having the best day in the world or you're sitting in the house and it's pouring of rain, you can't do anything, you don't speak to anybody or whatever. Suddenly, you get this ping on the message it takes your mind away from the problems. (TW1010, male, 57 years—Week 12 interview)

Most participants expressed that they had identified with quotes and examples from other people with strokes provided in the KATS messages, and highlighted that they took comfort and felt reassured by the quotes:

Yes, well it was interesting to see that you weren't the only one that had a stroke, you heard other people's views and what their problems were and what they were doing to overcome their problems, so, it was good. (GW1008, male, 67 years—Week 12 interview)

You would pass the message that somebody said, this [activity] worked for them when it's raining or whatever, they try walking up the stairs. I remember that one because I did that because it was pouring with rain. I had been on my bike, but there are only so many times you can sit on the bike. Then I think the ping message came and I look at it and it says—I don't say the name, David again, he tried walking up the stairs on a rainy day, so, I did it, 3 or 4 times I walked up and down the stairs. Don't get me wrong, I'm a little bit out of breath each time but it's fun. (TW1010, male, 57 years—Week 12 interview)

Although details about the sources and pseudonymised nature of the quotes and examples used in the messages were documented in the intervention materials and explained to all participants at the beginning of the intervention, there were indications that some participants interpreted the quotes and examples as though they were coming from other people taking part in the KATS intervention at the same time as them:

Most of the messages were coming across, it was like people with their own engine, like I'm going to go and exercise, I'm going to do this, I'm going to do that. (…) I found that most of the people who were texting seemed to have their own engine, as I call it, their own drive and they were going to make sure that they were going to do this and they were going to do that, which is what I was doing. (…) If you're one of these people who are going to wait for someone to say, come on, you should be doing this or, come on, you should be doing that. I know a stroke can make you depressed, it can do that, but the people who were texting you, all seemed to have, or the majority of them seemed to have this attitude. (GW1008, male, 67 years—Week 6 interview)

Similarly, although participants were informed at the beginning of the intervention that the KATS messaging system did not allow the research team to reply to any of their responses, most participants still chose to actively reply to text messages, with all but one of the participants sending text message responses at some point during the intervention.

Overall, our analysis suggested that participants generally understood the KATS intervention, were able to broadly describe it, and had a shared sense of its purpose:

I think at the start I was a bit apprehensive; it was like a step into the unknown really, I had no idea what it was going to entail. Certainly, going through the process, I found as we went on that things got better and I was more understanding of what it was going to entail (…) It's not a tailored programme for specific people, it's just basically, for individuals to find their own way as they go through the process because everyone will have their own goals and things they want to achieve individually. (TW1013, male, 56 years—Week 12 interview)

However, it became apparent from participants' descriptions and references to the nature of the intervention that the more nuanced, complex components of the KATS intervention were not relevant for all participants. For example, whilst the KATS text messages were theoretically informed and explicitly designed to go beyond acting as simple reminders, aiming to provide a structure that facilitated behaviour change, some participants still described the value and helpfulness of the intervention primarily as a reminder or trigger to physical activity:

The most useful thing is just to remind you, because sometimes you forget to do things, if there is nobody there to remind you, if your wife is not at home or you can forget, and you're watching TV or listening to records, you need a jolly along to say, it's about time you did some exercise, get up and move around for half an hour or whatever. So, the most useful aspect is a reminder. (TW1004, male, 61 years—Week 6 interview)

### 3.3 Making KATS work: Flexibility and tailorable guidance

Despite participants seeing the point of the intervention and considering it relevant and fitting in the context of their rehabilitation journey, it was unclear to what extent participants took advantage of the full range of behaviour change techniques offered by the intervention as ways to encourage increases in their activity levels. As expected for a remotely delivered self‐help intervention like KATS, there was variation in the amount of engagement with the range of behaviour change techniques offered by the intervention. This is illustrated by these two participants describing significantly divergent forms of engagement with the same intervention component, the calendar:

I've been using the calendar quite regularly the last few weeks, both for putting up things that are coming up, so, that you know what is going to be happening and also recording exercises that I've done, and I've found that quite rewarding. (GW1007, male, 70 years—Week 6 interview).

I'm afraid I don't even know where it [the calendar] is. It came in a brown envelope; it's still lying unopened. (TW1003, male, 69 years—Week 6 interview)

Participants found the messages to be understandable, helpful, interesting and relevant to guide their journey to recovery following discharge from therapy. Most participants made the intervention work by engaging with intervention components as suggested by the KATS messages and/or following more general suggestions or signposting included in the KATS messages. Some participants were able to link and tailor the guidance provided to meaningful tasks or get family members involved:

I just try to involve it [the hand affected by stroke] in everyday things, like taking hold of things, opening a door, putting on the shower and that sort of thing, and trying to consciously use the left hand [the hand affected by stroke] more for just doing things. That's one of the reasons I was keen to do the study, I wanted to try and keep these things going once the physio stopped. (GW1007, male, 70 years—Week 6 interview)

The motivational aspects embedded in the KATS messages connected well with most participants' mindset at this stage of rehabilitation/recovery, which was characterised by their determination to undertake everyday tasks and activities despite the perceived challenges and difficulty of staying active. Here, too, identification with others in a similar situation seemed to provide motivation and be a key driver of participants' commitment to continue to engage with the intervention. Participants highlighted how reading the quotes featured in the KATS messages and gaining insight into the experiences of other people with stroke had been motivating, with quotes and examples providing participants with role models for the behaviours they were seeking to engage with and embed into their lives:

It showed what they [other people with stroke] had done, and it helped me, I could do things that I thought I couldn't. (TW1009, female, 74 years—Week 6 interview)

### 3.4 Appraising the worth of KATS: Encouragement and friendliness

Participants valued being offered the opportunity to reflect on the physical activity they were doing at the time of receiving the text messages and consider the physical activity they could or should be doing:

I have read every message, I do sit and ponder on it and think about it and it does help me quite a lot and makes me think quite a lot of what is happening and what I should be doing and what have you. (GW1002, female, 60 years—Week 6 interview).

Other aspects of the messages, such as the terminology and tone used (e.g., relying on informal language and incorporating humour and trivia) were valued by participants. They appreciated the nonauthoritative, nonprescriptive nature of the KATS messages:

They [the messages] were quite friendly and not too formal, and I think that hits the right tone. You don't want to tell people to do things. You have to jog them along fairly gently. (TW1004, male, 61 years—Week 6 interview)

I think if you start moving down the formality route it's like you're bringing out the big stick or the wagging finger. You're giving instructions to people when you get formal as opposed to encouragement. (TW1003, male, 69 years—Week 12 interview)

Overall, regardless of the participants' level of engagement with the more detailed intervention components and activities, the KATS intervention was seen as worthwhile:

It's been like a good friend to me. (GW1002, female, 60 years—Week 12 interview)

I've found it very, very worthwhile. I've actually surprised myself how far I've come since starting the study (…) It's built up my confidence to actually attempt other things because I feel confident in that. (TW1013, male, 56 years—Week 12 interview).

Participants judged the KATS intervention to be advantageous for all stroke survivors following discharge from community‐based rehabilitation services. Perceived benefits extended beyond the element of support to promote physical activity, for example the affective/emotional effect of not feeling alone; the motivational aspect of monitoring progress; and softening the sharp ending of rehabilitation/therapy services at the point of discharge.

## 4 DISCUSSION

This qualitative study explored the views and experiences of the first cohort of participants to complete the KATS intervention about the meaning, engagement, workability and worth of the intervention. We found that participants were able to differentiate the new KATS intervention from current rehabilitation practice and perceived the benefits it could bring about for people with stroke. There was variation in the amount of engagement with the range of behaviour change techniques offered by the intervention, however, participants were able to tailor their experience of the intervention and make it work for them in different ways. Overall, participants saw the KATS intervention as worthwhile.

One key perceived benefit of the KATS intervention was its potential to address an unmet need experienced at the point of discharge from NHS‐funded therapy, which some participants associated with feelings of abandonment.^25^, ^26^ This important finding has already informed some intervention refinements. To ensure that the initial sense of abandonment is not echoed at the end of the KATS intervention, two additional weeks will be added at the end of the original 12‐week intervention cycle. During these two additional weeks, the frequency of message delivery will gradually decrease, and the focus will shift to preparing participants for the end of the intervention and maintaining engagement in activities.

The KATS intervention was perceived as both relevant and fitting in the context of participants' rehabilitation journeys. These perceptions were emphasised by the experiences during COVID‐19 restrictions, which brought about an increased sense of isolation that the KATS intervention helped address. This finding also helped demonstrate another important finding, that is, that some participants' understandings and perceived importance of the intervention were primarily tied to the very idea of receiving a text message (feeling seen, counted and supported) and were less about the content and components of the KATS intervention, which did not seem to matter as much to them as not feeling forgotten. Future research will further examine this finding with a view to establish whether it was disproportionally salient in our data due to the increased sense of isolation brought about by COVID‐19 restrictions.

Participants sense of feeling seen and supported is a valuable finding and a potentially important outcome for the KATS intervention to consider in a future trial. However, our analysis also showed that the more complex behavioural aims/components of the intervention were not relevant to all participants. Therefore, future research should investigate whether addressing participants' need for support following discharge might overshadow the full projected benefits of the intervention, that is, whether some participants' satisfaction with ‘just receiving’ text messages may also mean that they were not sufficiently receptive or motivated to invest thought and energy into changing their physical activity behaviour. If so, further intervention refinement may be warranted, and any future trials to evaluate the effectiveness of the intervention should reflect this in the set of primary and secondary outcomes to be measured.

These findings could help refine the intervention to support readiness for change in physical activity after stroke more comprehensively, although the KATS intervention was not designed to require a specific form or level of engagement from participants. The development of the intervention consciously adopted a nonprescriptive approach focusing on providing guidance, ideas and choices (rather than giving rigid instructions or directive messages) to allow for different forms and levels of engagement and tailoring of the intervention to individual circumstances, needs and preferences. This is an aspect that differentiates the KATS intervention from existing interventions in this area.^11^, ^12^, ^13^, ^14^ The range of ways to engage with the intervention were apparent in participants' experiences, from those more thoroughly committed to enact the full range of proposed intervention activities through to those making the intervention work for them by just engaging with general suggestions/signposting provided or linking their physical activity to meaningful everyday tasks. A nonprescriptive approach that acknowledges different forms of engagement and tailoring is in line with a person‐centred rehabilitation model^27^ and was an aspect of the intervention highly valued by participants, alongside other aspects of the messages' tone such as informality, humour and trivia.

One key aspect that shaped participants' positive experiences with the KATS intervention was the inclusion of quotes and examples from other people with stroke in the text messages. This was particularly meaningful to participants, providing them comfort and reassurance, with many noting they had identified with the quotes and examples included in the text messages. There were indications in our data that some participants seemed to interpret the quotes and examples as though they were coming from other people taking part in the KATS intervention at the same time as them. Therefore, it is important for future evaluation to better understand how people perceive these messages and whether further intervention refinement might be needed. This observation raises ethical implications about how successful the KATS intervention communication strategies were in providing transparent explanations about the source and nature of the quotes. Clear explanation using communication strategies relevant to this population, of whom many have cognitive and communication impairments, is, therefore, another important aspect of the KATS intervention that should continue to be ensured by this and other interventions with this population.

Similarly, despite clear and explicit communication to all participants that text messages were prewritten (rather than in real time), and that the KATS messaging system did not allow for the research team to reply to any of their responses, most participants still chose to actively engage with the text messages by replying to them. The ‘humanisation’ of text message‐based interventions has been described in other studies^28^ and can be explained by increasingly generalised perceptions of mobile phones as highly personal and emotionally significant objects.^29^, ^30^ Future research could explore any potential overlaps with peer support interventions for people with stroke^31^, ^32^, ^33^ and consider whether any of their mechanisms and outcomes (e.g., increasing knowledge and motivation, promote vicarious learning and problem‐solving, feelings of hope and validation, decreased sense of isolation) can help improve how text messaging interventions can better address the support needs of people with stroke.

Whilst the ‘humanisation’ of the intervention can be considered an example of positive engagement, it is important to ensure that future research/implementation strategies for these types of intervention continue to explicitly address the ethical implications involved in being transparent about the origin of the messages, particularly when delivered to populations for whom digital literacy might be a persistent barrier to equity.^34^

Our study has some limitations. The sampling strategy for this qualitative study had to rely on the feasibility study sampling and recruitment strategy, which meant that our sample was not as diverse and information rich as it would have been if we had been able to employ a purposeful sampling strategy. The feasibility study sample had limited variation on certain domains (e.g., only three participants were women; only three participants lived alone; and the Modified Rankin Score for nine participants was three). Future research should seek to recruit from a wider range of geographical areas and clinical settings to allow for a more robust and comprehensive qualitative sampling strategy, and a more in‐depth exploration of any issues of relevance to those specific domains for which our sample was limited.

## 5 CONCLUSIONS

Participants were able to differentiate the new KATS intervention from current rehabilitation practice, perceived it as relevant and fitting in the context of participants' rehabilitation journeys, and assessed it as worthwhile. There was variation in the amount of engagement with the range of behaviour change techniques offered by the intervention, but participants were able to tailor their experience of the intervention and make it work for them in different ways. Perceived benefits went beyond the element of promoting physical activity to include the emotional effect of not feeling alone, the motivational aspect of monitoring progress, and softening the sharp ending of rehabilitation/therapy services at the point of discharge. Future research will test the effectiveness of the KATS intervention in promoting physical activity and stroke recovery, including any associations with relevant social and emotional secondary outcomes, as well as explore more fully how social and behavioural mechanisms of action are experienced and enacted by participants.

## AUTHOR CONTRIBUTIONS

**Albert Farre:** Conceptualisation; formal analysis; methodology; supervision; writing—original draft; writing—review and editing. **Jacqui H. Morris:** Conceptualisation; formal analysis; funding acquisition; methodology; supervision; writing—review and editing. **Linda Irvine:** Conceptualisation; formal analysis; funding acquisition; investigation; methodology; writing—review and editing. **Stephan U. Dombrowski:** Conceptualisation; funding acquisition; methodology; supervision; writing—review and editing. **Jenna P. Breckenridge:** Conceptualisation; funding acquisition; methodology; supervision; writing—review and editing. **Gozde Ozakinci:** Conceptualisation; funding acquisition; methodology; supervision; writing—review and editing. **Thérèse Lebedis:** Conceptualisation; supervision; writing—review and editing. **Claire Jones**: Conceptualisation; funding acquisition; software; supervision; writing—review and editing.

## CONFLICT OF INTEREST STATEMENT

The authors declare no conflict of interest.

## Data Availability

Data are available on reasonable request. Access to data can be arranged by contacting the study's Chief Investigator, Dr Jacqui H Morris (j.y.morris@dundee.ac.uk) to discuss data sharing, data requirements and conflicts of interest, in line with UK and other regulations, including ethics approvals.
